# Supplementary material for: Predictability and persistence of prebiotic dietary supplementation in a healthy human cohort
Source: Sci Rep. 2018 Aug 23;8:12699. doi: 10.1038/s41598-018-30783-1 (PMC6107591; doi:10.1038/s41598-018-30783-1)
Supplement: Supplementary file 1 — Supplementary information [file 41598_2018_30783_MOESM1_ESM.doc]

Predictability and persistence of prebiotic dietary supplementation in a healthy human cohort

Supporting Information

**Authors:** Thomas Gurry^1-3^, HST Microbiome Consortium^4,5^, Sean M Gibbons^1-3^, Le Thanh Tu Nguyen^1,2^, Sean M Kearney^1,2^, Ashwin Ananthakrishnan^6^, Xiaofang Jiang^1-3^, Claire Duvallet^1,2^, Zain Kassam^1,7^, Eric J Alm^1-3†^*

**List of authors in the HST Microbiome Consortium:** Paul H. Dannenberg^4,5^, Samuel G. Finlayson^4,5^, Travis K. Hughes^4,5^, Claudio Macias-Trevino^4,5^, Kwadwo Owusu-Boaitey^4,5^, Andre Shomorony^4,5^, Suan Lian Tuang^4,5^, Max L. Valenstein^4,5^, Kathy K. Wang^4,5^, Michael Pei-hong Wu^4,5^, Travis I. Zack^4,5^

**Affiliations:**

^1^Department of Biological Engineering, Massachusetts Institute of Technology, Cambridge MA 02139

^2^Center for Microbiome Informatics and Therapeutics, Massachusetts Institute of Technology, Cambridge, MA 02139

^3^The Broad Institute of MIT and Harvard, Cambridge, MA 02142

^4^Harvard-MIT Program, Health Sciences and Technology, Cambridge, MA 02139

^5^Institute for Medical Engineering and Sciences, Massachusetts Institute of Technology, Cambridge MA 02139

^6^Division of Gastroenterology, Massachusetts General Hospital, Harvard Medical School, Boston, MA 02114

^7^OpenBiome, Somerville, MA 02143

*****Corresponding Author (ejalm@mit.edu) and Lead Contact

Figure S1. The same OTU time series as those shown in Fig. 1c, but with RDP taxonomy assignments shown explicitly.

Figure S2. Summed relative abundances of Archaeal OTUs belonging to the Methanobacteria class in subjects randomized to the cellulose arm. Only subjects A020, A034 and A079 had non-zero relative abundance of this Archaeal clade in any samples considered. All other subjects are depicted in greyscale, with no observable blooms.

Figure S3. (a) ROC curve for Random Forest Classifier for preB/post1 samples; AUC=0.93, *p*=8.72×10^-7^ (*N*=26 subjects). (b) ROC curve for Random Forest Classifier for preB/post2 samples; AUC=0.90, *p*=1.48×10^-6^ (*N*=23 subjects).

| **Feature** | **Feature importance** | **Mean abundance (baseline sample)** | **Mean abundance (post1 sample)** |
| --- | --- | --- | --- |
| *Lachnospiracea incertae sedis* (denovo84) | 0.033 | 0.00144 | 0.00017 |
| *Roseburia* (denovo4) | 0.027 | 0.05274 | 0.01227 |
| *Bifidobacterium* (denovo71) | 0.025 | 0.01251 | 0.00093 |
| Lachnospiraceae (g__; denovo151) | 0.024 | 0.00041 | 0.00000 |
| *Ruminococcus* (denovo39) | 0.019 | 0.00916 | 0.00029 |
| *Ruminococcus* (denovo40) | 0.019 | 0.00774 | 0.00005 |
| *Lachnospiracea incertae sedis* (denovo155) | 0.015 | 0.00091 | 0.00000 |
| *Lachnospiracea incertae sedis* (denovo65) | 0.015 | 0.00627 | 0.00320 |
| *Clostridium XI* (denovo157) | 0.014 | 0.00094 | 0.00073 |
| Lachnospiraceae (g__; denovo956) | 0.013 | 0.00007 | 0.00001 |

Table S1. Top 10 features, ranked by feature importance, from the Random Forest Classifier in Fig. S2a.

| **Feature** | **Feature importance** | **Mean abundance (baseline sample)** | **Mean abundance (post2 sample)** |
| --- | --- | --- | --- |
| *Faecalibacterium* (denovo1102) | 0.021 | 0.00005 | 0.00000 |
| Lachnospiraceae (g__; denovo151) | 0.021 | 0.00028 | 0.00000 |
| *Streptococcus* (denovo235) | 0.018 | 0.00006 | 0.00021 |
| Enterococcaceae (g__; denovo286) | 0.014 | 0.00003 | 0.00010 |
| *Streptococcus* (denovo141) | 0.014 | 0.00042 | 0.00169 |
| *Ruminococcus* (denovo40) | 0.011 | 0.00837 | 0.00120 |
| *Coprococcus* (denovo62) | 0.011 | 0.00116 | 0.00238 |
| *Dorea* (denovo26) | 0.011 | 0.00259 | 0.00786 |
| Lachnospiraceae (g__; denovo10) | 0.010 | 0.00703 | 0.01555 |
| Ruminococcaceae (g__; denovo136) | 0.009 | 0.00016 | 0.00034 |

Table S2. Top 10 features, ranked by feature importance, from the Random Forest Classifier in Fig. S2b.
